# Peripheral extracellular vesicles in neurodegeneration: pathogenic influencers and therapeutic vehicles

**DOI:** 10.1186/s12951-024-02428-1

**Published:** 2024-04-12

**Authors:** Xixi Liu, Lu Shen, Meidan Wan, Hui Xie, Zhenxing Wang

**Affiliations:** 1 Department of Orthopedics, Movement System Injury and Repair Research Center, Xiangya Hospital, Central South University, Changsha, Hunan 410008 China; 2 Department of Neurology, Xiangya Hospital, Central South University, Changsha, Hunan 410008 China; 3 Hunan Key Laboratory of Angmedicine, Changsha, Hunan 410008 China; 4 National Clinical Research Center for Geriatric Disorders (Xiangya Hospital), Changsha, Hunan 410008 China; 5 Engineering Research Center of Hunan Province in Cognitive Impairment Disorders, Changsha, Hunan 410008 China; 6 Hunan International Scientific and Technological Cooperation Base of Neurodegenerative and Neurogenetic Diseases, Changsha, Hunan 410008 China

**Keywords:** Neurodegenerative disease, Extracellular vesicles, Blood-brain barrier, Peripheral-brain axis, Therapeutic delivery

## Abstract

Neurodegenerative diseases (NDDs) such as Alzheimer’s disease, Parkinson’s disease, and amyotrophic lateral sclerosis epitomize a class of insidious and relentless neurological conditions that are difficult to cure. Conventional therapeutic regimens often fail due to the late onset of symptoms, which occurs well after irreversible neurodegeneration has begun. The integrity of the blood-brain barrier (BBB) further impedes efficacious drug delivery to the central nervous system, presenting a formidable challenge in the pharmacological treatment of NDDs. Recent scientific inquiries have shifted focus toward the peripheral biological systems, investigating their influence on central neuropathology through the lens of extracellular vesicles (EVs). These vesicles, distinguished by their ability to breach the BBB, are emerging as dual operatives in the context of NDDs, both as conveyors of pathogenic entities and as prospective vectors for therapeutic agents. This review critically summarizes the burgeoning evidence on the role of extracerebral EVs, particularly those originating from bone, adipose tissue, and gut microbiota, in modulating brain pathophysiology. It underscores the duplicity potential of peripheral EVs as modulators of disease progression and suggests their potential as novel vehicles for targeted therapeutic delivery, positing a transformative impact on the future landscape of NDD treatment strategies.

**Search strategy** A comprehensive literature search was conducted using PubMed, Web of Science, and Scopus from January 2000 to December 2023. The search combined the following terms using Boolean operators: “neurodegenerative disease” OR “Alzheimer’s disease” OR “Parkinson’s disease” OR “Amyotrophic lateral sclerosis” AND “extracellular vesicles” OR “exosomes” OR “outer membrane vesicles” AND “drug delivery systems” AND “blood-brain barrier”. MeSH terms were employed when searching PubMed to refine the results. Studies were included if they were published in English, involved human subjects, and focused on the peripheral origins of EVs, specifically from bone, adipose tissue, and gut microbiota, and their association with related diseases such as osteoporosis, metabolic syndrome, and gut dysbiosis. Articles were excluded if they did not address the role of EVs in the context of NDDs or did not discuss therapeutic applications. The titles and abstracts of retrieved articles were screened using a dual-review process to ensure relevance and accuracy. The reference lists of selected articles were also examined to identify additional relevant studies.

## Introduction

Neurodegenerative diseases (NDDs) epitomize a group of maladies that insidiously dismantle the central nervous system (CNS), heralding significant biomedical challenges in an aging population. These disorders, including Alzheimer’s disease (AD), Parkinson’s disease (PD), and amyotrophic lateral sclerosis (ALS), not only precipitate profound neurological impairment but also impart considerable socioeconomic strain as life expectancies elongate globally [1]. NDDs are typified by the inexorable loss of neuronal integrity and function, culminating in a spectrum of symptoms from cognitive decline to motor and muscular debilitation. Traditional paradigms attribute these phenotypes to an array of cellular malfunctions, including compromised mitochondrial efficacy, oxidative stress, and defective autophagic processes [2–6]. Yet, these canonical pathways do not wholly delineate the complexity of NDD pathologies, nor do they provide the panacea for their cure, suggesting that additional, intricate mechanisms are at play.

At the vanguard of emerging mechanisms are extracellular vesicles (EVs), nano- to micron-sized carriers, which have garnered attention for their role in mediating cellular communication beyond the confines of the CNS [7]. These vesicles, encompassing both exosomes (30–100 nm) derived from endosomal compartments and the more heterogeneous ectosomes (50–1000 nm), encapsulate a microcosm of the cellular interior, carrying nucleic acids, proteins, lipids, and metabolites across biological frontiers [8–10]. Their repertoire extends beyond mere waste disposal to orchestrating physiological and pathological processes, facilitated by their capacity to breach the blood-brain barrier (BBB) and to mediate crosstalk between the CNS and peripheral tissues [7, 8, 11–13].

**Fig. 1:**
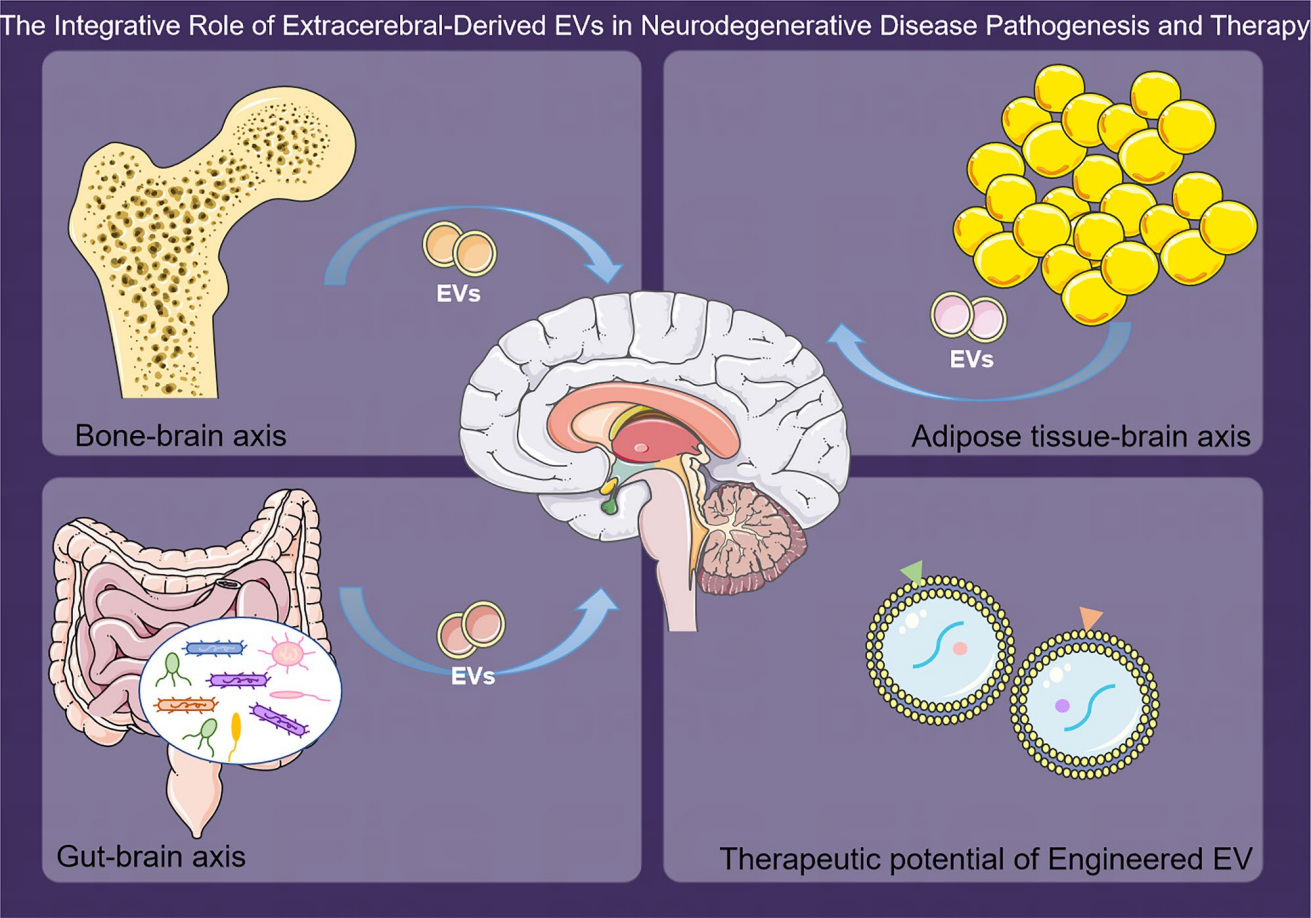
The integrative role of extracerebral-derived EVs in neurodegenerative disease pathogenesis and therapy. Extracerebral-derived EVs, originating from diverse peripheral sources such as bone, adipose tissue, and gut microbiota, are depicted as pivotal entities in the intercommunication between the periphery and the CNS. These vesicles possess the remarkable ability to traverse the systemic circulation, penetrate the BBB, and deliver molecular cargo that can influence NDD mechanisms. Additionally, the inherent biological characteristics of EVs present them as promising vectors for targeted therapeutic delivery to the CNS, offering a novel approach to treat NDDs. Engineered EVs, optimized for CNS targeting, could represent a paradigm shift in the modulation of pathogenic processes underlying NDDs and the administration of therapeutic agents

This review aims to elucidate the emerging narrative of extracerebral-derived EVs as dual agents: modulators of NDD progression and potential vectors for therapeutic intervention. By analyzing the influence of peripheral sources such as bone, adipose tissue, and gut microbiota, we aim to explain how EVs reflect and potentially initiate the molecular disruption that is characteristic of NDDs (Fig. 1). This review will traverse the landscape of recent discoveries that implicate EV-mediated peripheral-brain axis perturbations in the pathogenesis of AD, PD, and ALS. Furthermore, we will explore the broader implications of systemic illnesses that may potentiate neurodegeneration. The review will culminate in a discourse on the prospective utility of EVs in modulating brain pathology and their potential as novel therapeutic options for NDDs. In synthesizing these domains, our objective is to provide a comprehensive prospect that integrates the peripheral environmental factors with central pathologies, offering a novel lens through which to view and combat NDDs. By shedding light on the peripheral origins of EVs and their potential to traverse the BBB, we spotlight a frontier in NDD research for exploration and therapeutic innovation.

## Evidence of peripheral contributions to NDD Pathogenesis

The pathogenesis of NDDs has been predominantly attributed to CNS intrinsic factors, with hallmark neuropathological features such as amyloid-β (Aβ) plaques, hyperphosphorylated tau, and misfolded alpha-synuclein (α-syn) driving the research narrative [3]. This CNS-centric paradigm has overshadowed the potential contributory role of peripheral processes in the pathophysiology of these disorders. However, recent evidence challenges this brain-autonomous perspective, underscoring the influence of peripheral systems in both the production and aggregation of neurotoxic proteins within the CNS environment.

The interplay between the peripheral environment and the brain is emerging as a significant factor in the etiopathogenesis of NDDs. Peripheral cells have been shown to secrete exogenous toxic protein species that can spread to the CNS, potentially instigating or exacerbating neurodegenerative cascades [14]. Moreover, systemic inflammation and peripheral metabolic dysfunctions are increasingly recognized as key modulators of CNS pathology, suggesting that peripheral homeostatic alterations might be upstream events in NDD development [15]. The hypothesis that peripheral-derived factors contribute to NDDs extends beyond the simple translocation of pathogenic proteins. It encompasses a more complex scenario where peripheral dysregulation, mediated through EVs and other molecular conduits, orchestrates a detrimental cross-talk that may precipitate or perpetuate neurodegeneration [16]. These insights into the peripheral origins of brain pathology represent a paradigm shift, implicating a bi-directional axis of communication between the CNS and peripheral tissues that could underlie the progression of NDDs.

Consequently, the implication of the periphery in NDDs is reshaping our understanding of these diseases. This realization opens novel avenues for the identification of peripheral biomarkers and presents a frontier for therapeutic interventions that extend beyond the confines of the CNS. The investigation into peripheral contributions thus holds the potential to redefine therapeutic strategies, emphasizing the importance of a systemic approach in combatting neurodegeneration.

### Alzheimer’s disease and systemic biological interactions

AD, characterized by a profound decline in cognitive faculties and the most prevalent form of dementia, presents a formidable challenge to the medical community [17]. The histopathological signatures of AD include the extracellular deposition of Aβ peptides and the intracellular aggregation of hyperphosphorylated tau protein [18]. Despite extensive research, therapeutic strategies targeting these neuropathological features, specifically aimed at the clearance of Aβ or tau, have not yet resulted in significant clinical benefit [19]. This therapeutic impasse has prompted a reassessment of the etiopathogenic mechanisms underlying AD.

A seminal advance in this context is the growing appreciation of the systemic nature of AD, recognizing that the disease may not solely originate within the brain’s precincts. It has been posited that peripheral cells are capable of producing Aβ, which could instigate or exacerbate cerebral amyloidosis [20, 21]. This was compellingly demonstrated in a murine model where the transplantation of bone marrow cells from transgenic mice harboring AD-associated mutations into wild type (WT) counterparts resulted in the manifestation of AD-like neuropathology and cognitive deficits [22].

Moreover, the biological interplay between the periphery and the CNS is increasingly implicated in the progression of AD pathology, particularly through the roles of EVs. EVs, as carriers of intercellular communication, have been identified in the plasma of AD patients at elevated levels. They transport not only the secretases involved in APP processing but also an array of inflammatory mediators [23, 24]. This compelling evidence suggests a mechanism whereby systemic EVs may actively contribute to the neuropathological cascade of AD. Furthermore, peripheral organs exposed to aging, metal ion dysregulation, or infectious stimuli may launch an inflammatory response and release protease-rich EVs into the circulatory system. These EVs may traverse the BBB via the choroid plexus, becoming inadvertent couriers of pathogenicity to the hippocampal neurons, the epicenter of memory formation in the brain. Additionally, it has also been observed that circulating EVs in AD patients can disrupt the integrity of the BBB by diminishing the expression of vascular endothelial-cadherin, a key adherens junction protein [25]. This perturbation of the BBB may potentiate the influx of pathogenic molecules into the brain parenchyma, further fueling the neurodegenerative process.

In light of these findings, the relationship between systemic biology and AD emerges as a complex web of interactions, with implications that transcend the brain’s boundaries. By recognizing the peripheral contributions to AD, a new perspective is fostered, one that views AD not merely as a disorder of the brain but as a reflection of systemic imbalance. This systemic perspective could herald novel diagnostic markers and therapeutic targets, ultimately reshaping the strategies for combating this pervasive disease.

### Parkinson’s disease: beyond the central Dogma

PD, the senescence-associated scourge of the dopaminergic system, ranks as the second most prevalent neurodegenerative disorder among the elderly [26]. The pathognomonic feature of PD is the presence of Lewy bodies, and neuronal cytoplasmic inclusions predominantly composed of misfolded α-synuclein (α-syn), which mark the neuronal landscape of the disease [27]. The classical view of PD as a disease confined to the CNS has been challenged by revelations of widespread α-syn deposition, not just in the brain but also in peripheral tissues including the intestine, pancreas, heart, salivary glands, and skin [28–30].

The transmissibility of α-syn pathology represents a paradigm shift in understanding the pathogenesis of PD, where EVs emerge as potential vectors for the dissemination of pathological proteins. Recent studies have implicated EVs as a mechanism for the propagation of α-syn, suggesting a prion-like model of spread within and possibly beyond the confines of the CNS [31, 32]. The role of inflammation, an ever-present specter in neurodegenerative conditions, is now being reinterpreted in the context of PD. There is accumulating evidence that neuroinflammation, arising peripherally, can potentiate central inflammatory responses, leading to the activation of microglia and atrophy of astrocytes. This cascade not only accentuates the neurodegenerative process but also magnifies the deleterious impact of α-syn oligomers, further compromising neuronal integrity and amplifying motor and non-motor deficits in PD models [33].

The involvement of peripheral EVs as conduits for inflammatory signals provides a mechanistic link between systemic inflammation and neurodegeneration. Experiments demonstrating that blood-transformed EVs from aged mice can provoke glial activation in the brains of younger recipients underscore the profound influence of systemic factors on central pathologies [34]. Moreover, EVs derived from inflamed macrophages (Mϕ) have been shown to contribute to the progression of PD pathology, intensifying neuroinflammation, and exacerbating disease pathology [35]. This body of evidence propels the notion that PD is not an isolated central phenomenon but a systemic ailment with significant peripheral contributions.

### Amyotrophic lateral sclerosis and peripheral system cross-talk

ALS, an inexorably progressive neurodegenerative disorder, induces motor neuron demise, leading to pervasive muscle weakness and ultimately, respiratory failure [36]. Central neuroinflammation is recognized as a cardinal pathological feature, contributing to the relentless neuronal attrition observed in ALS [37]. Notably, the neuroinflammatory cascade in ALS is not isolated to the CNS; it involves a dynamic interplay with systemic immune components. Proinflammatory cells such as monocytes and T lymphocytes, along with their secretory immunoreactive molecules, have been implicated in disease exacerbation, indicating a substantial peripheral contribution to disease dynamics [38–40].

The peripheral immune signature of ALS patients has been found to be distinctly aberrant, with an altered monocyte distribution that not only reflects systemic immune dysregulation but also correlates with their capacity to breach the CNS barriers and propagate neuroinflammation [38]. This is further compounded by documented perturbations in the integrity of the CNS barriers in both ALS rodent models and patients, which potentiate the ingress of noxious peripheral factors into the neural environment [41].

EVs are emerging as pivotal entities in the molecular crosstalk between peripheral systems and the CNS in ALS. They are suspected couriers of pathogenic cargo, with a putative role in the dissemination of neurotoxic elements toward motor neurons (MNs) [40, 42]. A recent finding accentuates the toxic impact of skeletal muscle-derived EVs from sporadic ALS patients on MNs, underscoring the direct pathogenic conduits between affected muscles and neuronal cells [43]. Additionally, the therapeutic potential of EVs has been glimpsed in studies where EVs from peripheral mesenchymal stromal cells exert immunomodulatory effects, possibly mediated by microRNAs targeting microglial cells, thus offering a beacon of hope in the modulation of neuroinflammation in ALS [44].

In summary, the evidence delineating the role of peripheral factors in the pathogenesis of NDDs is compelling, warranting a re-evaluation of traditional CNS-centric models. By incorporating a holistic view that integrates systemic physiology with neuropathology, we stand on the cusp of unveiling the multifactorial nature of these complex disorders, potentially heralding a new era in their management.

## Deciphering the influence of bone-derived EVs on neurodegeneration

The skeletal system, an often-overlooked contributor to neurodegenerative pathology, dispatches EVs into the peripheral circulation that bears the capacity to influence brain health [45]. Bone-derived EVs, encompassing those from bone marrow stem cells, immune cells, and osteocytes, are increasingly recognized for their role in modulating neural environments, with potential implications in the pathogenesis of NDDs. In the nexus between bone health and neurodegeneration, current research delineates a neuroprotective axis steered by the release of EVs from healthy bone marrow stem cells. These EVs are armed with regenerative cargo, growth factors, anti-inflammatory cytokines, and neurotrophic molecules, that endorse neuronal survival and plasticity [46, 47]. The dichotomy in the role of bone marrow immune cell-derived EVs is stark; while some subsets foster neuroprotection and repair, others may convey pro-inflammatory signals, exacerbating neurodegenerative processes [48, 49].

Recent epidemiological threads weave a compelling narrative; diseases deteriorating bone integrity, such as osteoporosis (OP) and osteoarthritis (OA), are correlated with an augmented risk of AD and PD [47, 50–54]. This interrelationship hints at a scenario where diseased bone could pivot from a sanctuary to a source of pathological EVs. Dysfunctional osteocyte-derived EVs, in the environment of diseases such as OP, may lose their neuroprotective attributes or, worse, transport pathological molecules that could precipitate or aggravate NDDs [47].

### Osteo-neural crosstalk: EVs from bone marrow stem cells

The intercommunication between the bone marrow niche and the CNS is a complex and bidirectional dialogue, in which bone marrow-derived stem cells (BMSCs) including hematopoietic stem cells (HSCs), mesenchymal stem cells (BM-MSCs), and endothelial progenitor cells (EPCs), play pivotal roles. These cells are endowed with the remarkable plasticity to self-renew and differentiate into various cell types, thus standing at the forefront of regenerative medicine and neuroprotection [55, 56]. HSCs are instrumental in CNS repair mechanisms, primarily through trans-differentiation processes. Long-standing evidence intimates a compromised regenerative capacity in the context of NDDs; specifically, an attenuated hematopoietic supplement is observed in patients with AD and PD [57, 58]. This insufficiency predicates a deleterious impact on the intrinsic repair capabilities of CNS. BM-MSCs have garnered substantial attention due to their therapeutic potential in neurodegenerative disorders. Notably, intravenous administration of BM-MSCs has been shown to mitigate pathological alterations and foster neurogenesis within murine models of AD and PD, evincing their regenerative abilities [55, 59, 60]. Furthermore, the intrathecal introduction of these cells in a rodent model of ALS has led to significant disease modification, enhancing motor function and prolonging lifespan [61].

The therapeutic effects of BMSCs may be attributed, at least in part, to the EVs they secrete. These EVs possess the functional components derived from their progenitor cells, carrying inherent targeting properties and the capacity to transverse biological barriers [62, 63]. In AD murine models, BM-MSC-derived EVs, upon intracerebral administration, have exhibited efficacy in reducing Aβ plaque burdens and neuronal atrophy, notably within the cerebral cortex and hippocampus [64]. These vesicles carry the upregulation of brain-derived neurotrophic factor (BDNF) and neprilysin (NEP), a critical Aβ-degrading enzyme, simultaneously attenuating neuroinflammatory processes [62, 65]. Human BM-MSC-derived EVs have further demonstrated their capacity to induce differentiation in neural progenitor cells and have yielded beneficial outcomes in PD animal models, enhancing both motor function and histological integrity [66]. Additionally, EPC-derived EVs have been documented to interface with brain endothelial cells, promoting the restoration of the compromised blood-CNS barrier in ALS models [67, 68].

### Immuno-regulatory role of bone marrow immune cells-derived EVs

The bone marrow serves as a reservoir for the immune cells, producing a diverse array of components integral to the function of immune system [69]. Among these, EVs derived from bone marrow immune cells have emerged as crucial mediators of intercellular communication, influencing both physiological and pathological processes, including neurodegeneration. Bone marrow immune cells, encompassing a broad spectrum from myeloid progenitors to mature monocytes and lymphocytes, actively secrete EVs that participate in the regulation of immune responses [49]. These vesicles carry a payload of bioactive molecules, including cytokines, growth factors, and nucleic acids, that can modulate immune activity within the CNS [70]. The perturbation of EV signaling may contribute to the pathogenic processes underlying NDDs, suggesting a potential therapeutic target or biomarker for disease progression.

The crosstalk between the peripheral immune system and the CNS is a critical component of neuroinflammatory responses in neurodegeneration [71, 72]. EVs from bone marrow immune cells can cross the BBB, influencing the CNS environment. They are implicated in various mechanisms, such as modulating the activation state of microglia, the resident immune cells of the brain, which have been associated with the pathogenesis of diseases like multiple sclerosis (MS) and AD [73, 74]. The pathophysiological relevance of myeloid-derived EVs in NDDs is underscored by their capability to influence neuroinflammation. In models of AD, for instance, myeloid-derived EVs have been shown to enhance the phagocytic activity of microglia towards Aβ aggregates, indicating a potential for therapeutic modulation of innate immune responses [75].

As biomarkers, bone marrow-derived EVs hold promise for the early diagnosis and monitoring of NDDs. Their presence and alterations in their molecular cargo in the peripheral blood may reflect CNS pathology, allowing for less invasive assessments of disease state and progression [70]. Future studies must aim to elucidate the precise molecular mechanisms governing EV-mediated interactions between the bone marrow and CNS to harness their full therapeutic and diagnostic potential.

### Osteocytes as remote regulators of CNS health

Osteocytes, the chief architects of the bone microenvironment, emerge as unconcerned conductors in the maintenance of CNS health, underscoring a novel paradigm in neurobiology. These cells, constituting up to 95% of the bone matrix populace, boast remarkable longevity, with their lifespan extending over two decades, echoing the protracted durability of neurons. Osteocytes extend an intricate network of dendritic processes (on average 50), similar to neuronal dendrites, facilitating intercellular communication through gap junctions and canalicular conduits within the mineralized bone matrix [76]. Historically relegated to the role of mechanical placeholders within bone, osteocytes are now recognized as dynamic endocrine players, orchestrating bone remodeling and mineral metabolism [77]. Beyond their traditional confines, osteocytes reach into systemic physiology through their secretome, influencing distal organ functions [76, 78, 79]. Their ability to dispatch EVs containing bioactive molecules underscores a remote regulatory capacity that extends to the CNS.

Emergent evidence delineates the role of osteocyte-derived EVs as conveyors of neuroprotection. Our investigations unveiled that osteocytes harvested from young mice secrete EVs that potentiate the mitigation of cognitive decline and interfere with the pathological cascade of AD [47]. These findings pivotally suggest that osteocytes, through their secreted EVs, may exert a salutary influence on the CNS, providing a potential therapeutic option. The age-related decline in the regenerative capacity of osteocyte-derived EVs reflects a parallel with neurodegenerative progression, suggesting a senescence-associated diminishment in their neuroprotective efficacy. The characterization of osteocyte-derived EVs from young versus aged sources reveals differential molecular signatures, indicating a potential shift from neurotrophic to neuroinflammatory profiles with aging. This posits osteocyte-derived EVs as both biomarkers and modifiable entities in the context of neurodegeneration. Investigations into the mechanistic pathways engaged by osteocyte-derived EVs highlight their role in modulating neuroinflammatory responses, amyloidogenic pathways, and synaptic plasticity. Through the delivery of specific proteins and signaling molecules, these vesicles may attenuate neuroinflammatory cascades and modulate Aβ metabolism, offering insights into the crosstalk between skeletal health and cognitive function.

Recognition of osteocytes as peripheral regulators of CNS health via EV-mediated signaling has revealed a previously underappreciated dimension of the bone-brain axis. The prospect of leveraging the neuroprotective potential of osteocyte-derived EVs opens new avenues for therapeutic intervention in NDDs, potentially altering the trajectory of conditions such as AD. As the research landscape evolves, the imperative to decode the molecular language of osteocyte-derived EVs will be paramount in translating these findings into clinical reality.

**Fig. 2:**
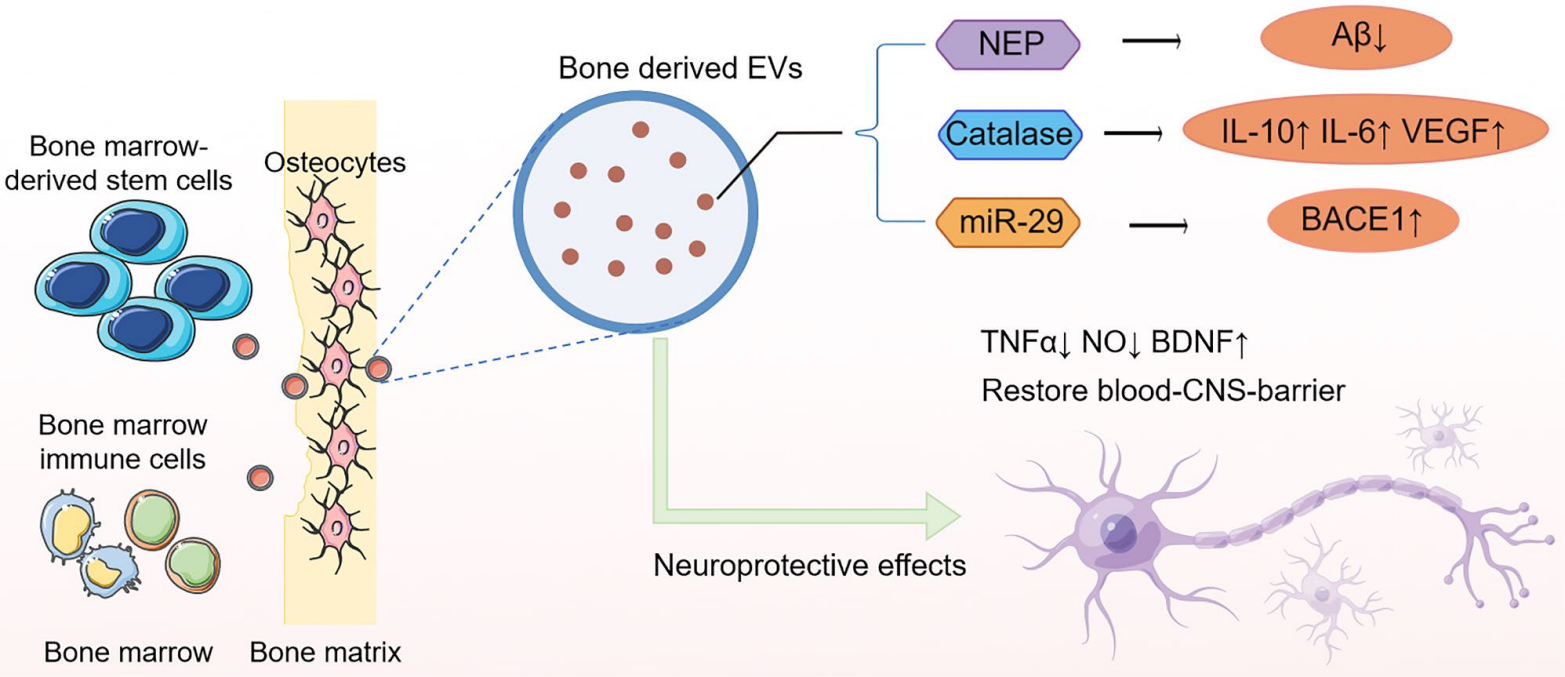
Deciphering the influence of bone-derived EVs on neurodegeneration. Bone derived EVs serve as important messengers in the bone-brain axis. EVs derived from bone marrow stem cells, immune cells, and osteocytes carry an array of functional molecules, including various proteins and miRNAs, which are implicated in neuroprotective mechanisms. These benefits are reduced when cells in the bones die or become inactive due to aging or disease states

In summary, emerging evidence suggest that bone derived EVs as important messengers within the bone-brain axis, integral to the maintenance of cerebral integrity (Fig. 2). These vesicular entities, dispatched by various cellular constituents of the bone, including marrow stem cells, immune cells, and osteocytes, harbor the capacity to transduce signals conducive to neuroprotection and cognitive function preservation. However, it is observed that the neuroregulatory potential of bone-derived EVs declines in the face of osteocellular senescence or pathological states such as OP, aligning with an increased vulnerability to NDDs. The attrition of osteocellular vitality, whether through apoptotic demise or dormancy, appears to coincide with a decline in the supportive secretome, implicating bone health as a critical factor in the etiopathogenesis of NDDs [80–82]. This inverse correlation between the robustness of bone-derived EVs and the incidence of disorders such as AD and PD illuminates the nuanced interplay between systemic skeletal conditions and neural degeneration. As such, osteopathy may not merely coexist with neurodegeneration but may actively precipitate its onset and progression, underscoring a need to re-evaluate therapeutic targets within this axis.

## Adipose tissue-derived EVs: messengers of metabolic and neurologic health

The interplay between adipose tissue and the CNS extends far beyond mere energy storage, engaging in a complex dialogue critical for maintaining metabolic and neurologic homeostasis [83]. As the primary energy reservoir and an endocrine powerhouse, adipose tissue orchestrates a symphony of hormones and bioactive molecules, profoundly influencing peripheral and central processes [84]. This section elucidates the dualistic nature of adipose tissue-derived EVs as conveyors of both metabolic information and neuropathological consequences.

Dysfunction in adipose tissue, marked by altered secretion of adipokines such as leptin and adiponectin, emerges as a pivotal factor in NDD etiology [85–88]. However, despite long-term intake of hypercaloric nutrition, the energy status of brain is lowered in obese patients [89]. The paradox of decreased cerebral energy availability in the face of hypercaloric intake, as observed in obesity, underscores a metabolic disconnect that extends to compromised neurotrophic support. Notably, diets rich in fats and sugars precipitate declines in brain-derived neurotrophic factor (BDNF) expression [90, 91], which is essential for neuronal survival and synaptic integrity. Proinflammatory adipokines further exacerbate neuroinflammation, disrupting CNS homeostasis [88, 92]. In this inflammatory environment, adipose tissue-derived EVs serve as both carriers of crisis and harbingers of healing. These vesicles, harnessing the intrinsic communicative properties of adipocytes, disseminate signals that can be pathogenic or protective, depending on their molecular cargoes and the metabolic context from which they originate [93].

Among the reparative pathways activated by adipose-derived signals, MSCs within adipose depots have garnered attention. Adipose tissue-derived MSCs (AD-MSCs) and their EVs exhibit pronounced neuroprotective effects [94–98]. In vitro, EVs from human AD-MSCs (hAD-MSCs) mitigate microglial activation and inflammatory cytokine release, simultaneously enhancing anti-inflammatory mediator expression [96]. Moreover, in animal models of ALS, AD-MSC-derived EVs home to affected neural regions, attenuating motor neuron degeneration and glial activation [97].

However, the pathogenic potential of adipocyte-derived EVs surfaces in the context of obesity and diabetes. EVs from such compromised states can induce cognitive decline and synaptic deficits in otherwise healthy recipients, implicating miRNAs like miR-9-3p in the dysregulation of neuronal insulin signaling [99]. Furthermore, adipocyte EVs isolated from AD patients carry miRNAs (for example, miR-6760-3p and miR-6798-3p) that may perturb the cyclic AMP response element binding protein (CREB) signaling pathway in neurons, pivotal for memory and synaptic plasticity [100].

**Fig. 3:**
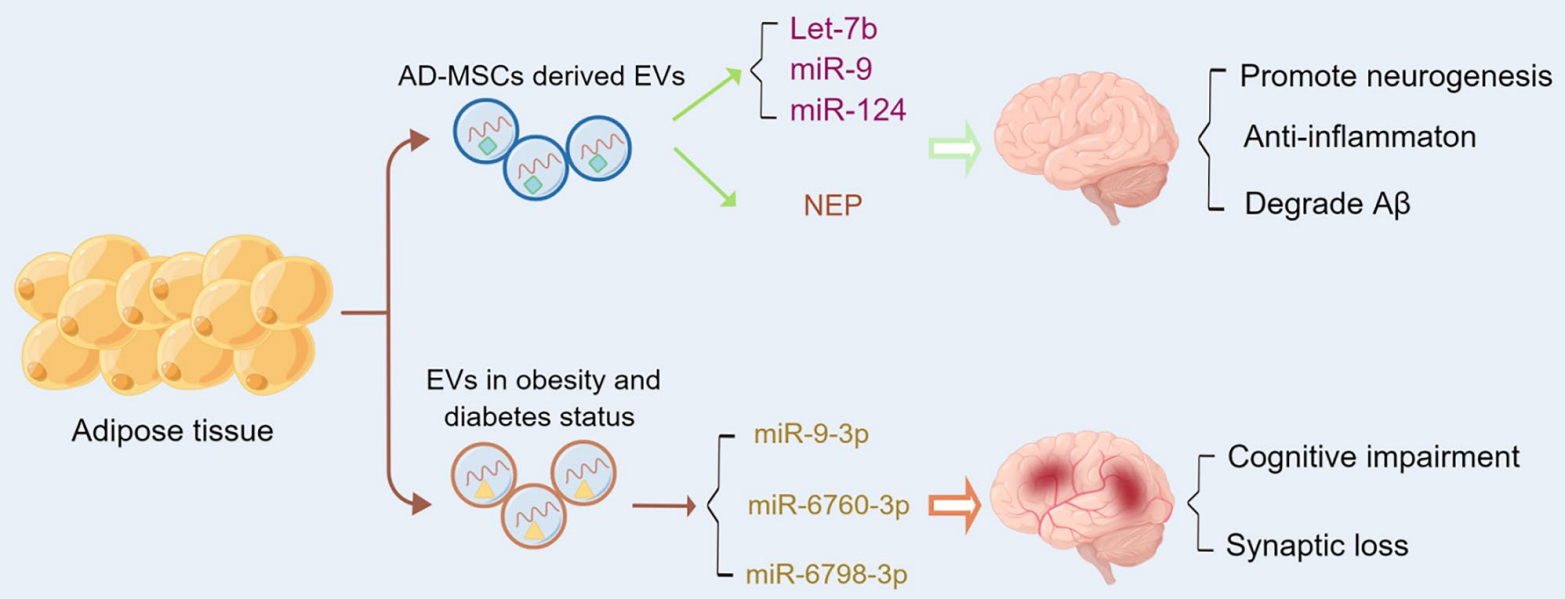
Adipose tissue-derived EVs: messengers of metabolic and neurologic health. EVs deriving from adipose tissue present the potential to mediate both neuroprotective and neuropathogenic interactions. In the upper panel, EVs released from healthy adipose tissue are rich in beneficial adipokines, anti-inflammatory cytokines, and growth factors that collectively support neurogenesis, synaptic plasticity, and cognitive function. In the lower panel, in the metabolic disorders pathological state, adipose tissue becomes a source of EVs laden with proinflammatory adipokines, dysregulated miRNAs, and other metabolic by-products that can impair cognitive function

In summary, adipose tissue-derived EVs constitute a crucial nexus between metabolic and neurologic health. In a state of physiological equilibrium, they transport molecules that uphold cognitive function and neural resilience. Conversely, metabolic dysregulation yields EVs laden with deleterious factors that propagate neuroinflammatory and degenerative pathways. Figure 3 delineates this duality, emphasizing the consequential role of adipose-derived signals in the integrity of the CNS.

.

## Gut-brain axis: microbiota-derived EVs as modulators of neural integrity

The intricate ecosystem of the human gut microbiota is a pivotal contributor to homeostasis, extending its influence beyond the gastrointestinal tract to modulate immune, neurological, and metabolic functions [101]. The gut-brain axis (GBA) epitomizes this bidirectional communication, with the gut microbiota acting as a key mediator [102, 103]. Disturbances in the microbiota, or dysbiosis, triggered by factors like antibiotics, stress, or infection, disrupt this communication and may lead to a permeabilized gut barrier, allowing for the systemic dissemination of pro-inflammatory agents [104].

Emerging evidence underscores the relationship between microbial dysbiosis and NDD pathogenesis, linking microbial imbalance to CNS anomalies, such as heightened BBB permeability, neuroinflammation, and accumulation of neurotoxic proteins [102, 105]. This imbalance in homeostasis can lead to infection by opportunistic pathogenic bacteria and increased BBB permeability, thus promoting neuroinflammation and the accumulation of neurotoxic misfolded proteins in the CNS [106–109]. Conversely, probiotic treatments have shown promising results. Strains of *Lactobacillus* and *Bifidobacterium*, for instance, have been noted to ameliorate cognitive deficits in AD models [110, 111], and *Akkermansia muciniphila* has demonstrated potential in alleviating symptoms in neurodegenerative mouse models [112, 113]. Probiotics can also help patients with PD to result in beneficial impacts on movement disorders and constipation [114].

Of particular interest are EVs secreted by gut bacteria. These EVs are similar to yet distinct from, mammalian exosomes and ectosomes, as they form through the budding of the bacterial outer membrane [115, 116]. These bacterial EVs have emerged as novel communicators within the GBA, traversing to the host brain via circulatory or neural pathways [117, 118]. For instance, *Helicobacter pylori* (*HP*) EVs can breach the BBB and provoke neuroinflammation through mechanisms involving glial cell activation and complement pathway signaling, potentially exacerbating amyloidogenic pathology [119, 120]. Moreover, neurotoxic EVs from pathogens, like *Pseudomonas aeruginosa, Paenalcaligenes hominis*, and *Aggregatibacter actinomycetemcomitans*, contain components such as lipopolysaccharides (LPS) that can invoke a robust immune response in the CNS [115, 121, 122]. In stark contrast, EVs from commensal bacteria with probiotic characteristics offer therapeutic promise. EVs from *Lactobacillus plantarum*, for example, have elicited antidepressant effects and counteracted amyloid-beta induced neuropathology in experimental models [123, 124].

**Fig. 4:**
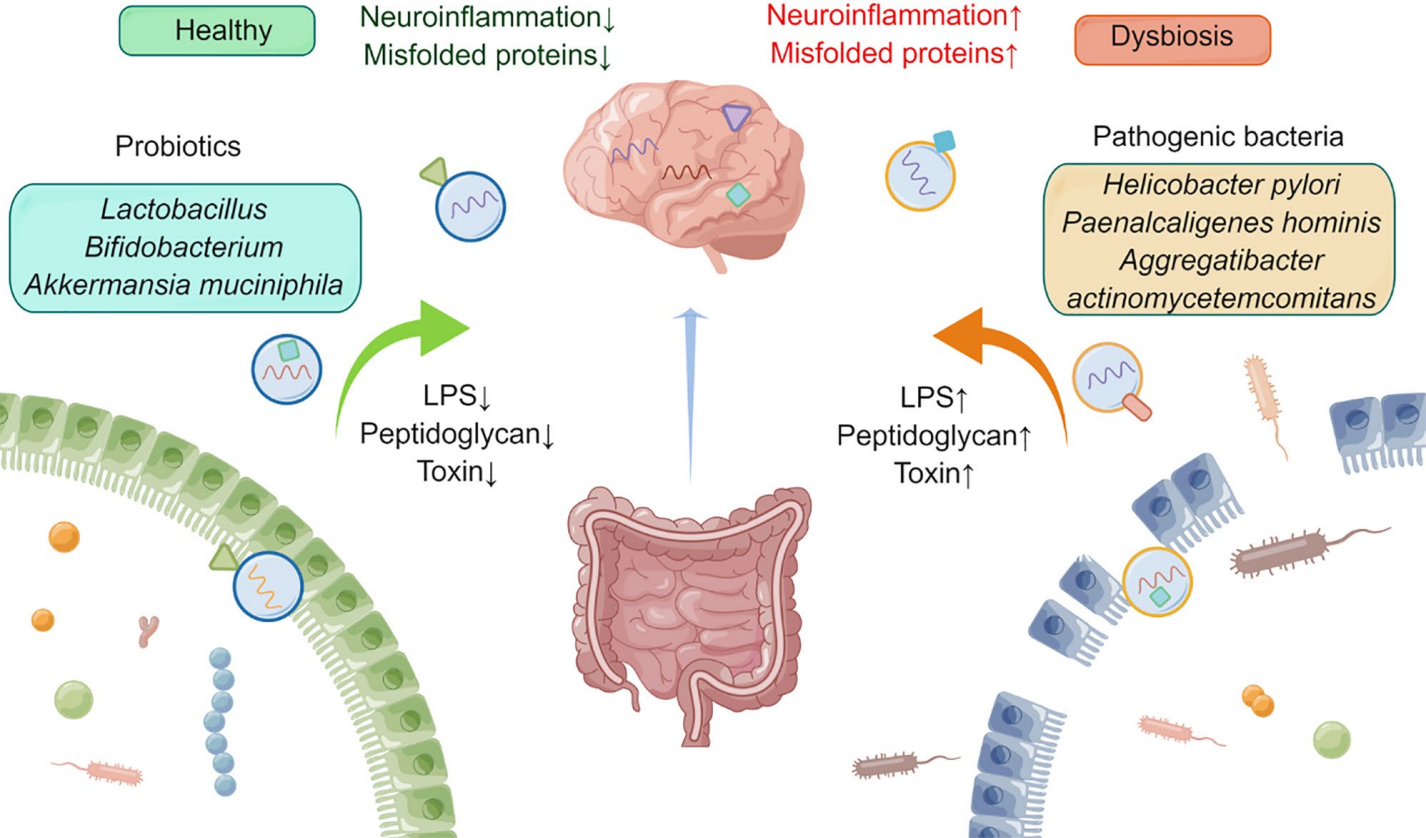
Gut-brain axis: microbiota-derived EVs as modulators of neural integrity. This schematic illustrates the dichotomous influences of EVs derived from gut microbiota on neural health. Probiotic EVs, originating from beneficial commensals, confer neuroprotective effects, which may ameliorate NDD pathology. Conversely, disruption of gut homeostasis allows pathogenic bacteria to release EVs that can translocate across the intestinal and the BBB. These vesicles transport inflammatory components, such as LPS, peptidoglycans, and bacterial toxins, into the CNS, potentially inducing immune responses that precipitate neuroinflammation and the aggregation of neurotoxic proteins

Thus, the modulation of the intestinal microbiota, favoring beneficial over pathogenic bacteria through dietary or pharmacological interventions, may be a viable strategy for preserving neural integrity. Probiotic-derived EVs, in particular, present an innovative avenue for therapeutic exploration, potentially offering a means to restore or maintain neurological health through the harmonization of the GBA. The clinical translation of these findings could revolutionize treatment paradigms for neurodegenerative disorders, providing a novel class of biologics derived from the gut microbiota itself (Fig. 4).

Overall, the effects of extracerebral-derived EVs on NDDs have a dual nature and can be modulated through various mechanisms. Table 1 provides a summary of the roles of different sources of extracerebral-derived EVs in NDDs, emphasizing their diverse functions and impacts on the pathogenesis of these disorders.

**Table 1:** Essential functions of EVs and their impact on NDDs

Original organs	Original cells	Cargo	Potential effects	Biological function	Reference
Bone-derived EVs	BM-MSCs	NEP, miR-29, Catalase, SphK/S1P	Beneficial	Increase BDNF, activate SphK/S1P signaling, degrade Aβ	[62, 64, 65]
	BM-EPCs	Angiogenic factors	Beneficial	Repair damaged microvascular endothelium	[67]
	BM-immune cells	Unknown	Beneficial	Degrade Aβ	[70]
	Osteocytes	Aβ degradation and mitochondrial energy metabolism factors	Beneficial	Ameliorate cognitive impairment, degrade Aβ	[47]
	BM-immune cells	Unknown	Detrimental	Produce Aβ oligomers, neuroinflammation	[73, 74]
Adipose tissue-derived EVs	AD-MSCs	NEP, let-7b, miR-9, miR-124	Beneficial	Degrade Aβ, anti-inflammation, neuroprotection	[94, 96, 97, 125]
	Adipocyte	miR-9-3p, miR-6760-3p, miR-6798-3p	Detrimental	Downregulate BDNF, downregulate CREB signaling	[99, 100]
Microbiota-derived EVs	*Lactobacillus*, *Bifidobacterium*, *Akkermansia muciniphila*	Unknown	Beneficial	Increase BDNF, upregulate MeCP2 and Sirt1	[123, 124]
	*Helicobacter pylori*	LPS, peptidoglycan, toxin	Detrimental	Activate C3-C3aR signaling, induce neuroinflammation	[119, 120]
	*Pseudomonas aeruginosa*, *Paenalcaligenes hominis*, *Aggregatibacter actinomycetemcomitans*	LPS, peptidoglycan, toxin	Detrimental	Induced cognitive impairment	[121, 122]

*Abbreviations*: MSCs, mesenchymal stem cells; BM-MSCs, bone marrow-derived MSCs; NEP, neprilysin; SphK/S1P, sphingosine kinase/sphingosine-1-phosphate; BDNF, brain-derived neurotrophic factor; Aβ, amyloid-β peptides; BM-EPCs, bone marrow endothelial progenitor cells; AD-MSCs, adipose tissue-derived MSCs; CREB, cyclic AMP response element binding protein; Mecp2, methyl-CpG binding protein 2; Sirt1, sirtuin 1; LPS, lipopolysaccharides; C3-C3aR, complement component 3-C3a receptor

## Therapeutic horizons: harnessing peripheral EVs for NDD intervention

In the vanguard of regenerative medicine and the therapeutic landscape for NDDs, EVs stand out as a promising cell-free modality, circumventing the complexities and ethical quandaries often associated with stem cell transplantation and other cell-based interventions [126]. These naturally occurring nanocarriers harbor the intrinsic ability to encapsulate and convey bioactive molecules—proteins, lipids, and nucleic acids—reflective of their cells of origin, thus preserving the potential for therapeutic modulation [127]. Their structural bilipid membrane composition not only ensures the integrity of their molecular cargo but also facilitates their navigation across the biological moat that is the BBB, a feature that is indispensable for the targeted delivery of therapeutics to the CNS [128].

The distinctive characteristic of EVs to serve as vectors for intercellular communication positions them as uniquely suited for the conveyance of reparative signals to neural tissues beleaguered by degenerative processes. In this paradigm, the horizon of NDD intervention is broadening, with EVs emerging as not only vehicles for direct therapeutic action but also as agents that can modulate the neuroinflammatory environment that is a hallmark of neurodegeneration. Moreover, the capacity of EVs to be engineered for enhanced targeting provides a platform for precision medicine, wherein modifications to EV surface proteins or contents can be tailored to address specific pathophysiological conditions within the CNS.

This section delineates the therapeutic potential of peripheral EVs, exploring the frontiers of their application in NDDs and discussing the mechanistic underpinnings that position these entities as a novel class of therapeutic agents. We will scrutinize the emerging evidence for their capability to modulate disease pathways, deliver pharmacological agents across the BBB, and initiate reparative processes within the diseased CNS. By harnessing the innate properties of EVs, and through innovative bioengineering strategies, there is a tantalizing prospect for the development of novel therapeutics that can navigate the challenges presented by the complexity of the human brain and the diseases that afflict it.

### Navigating the BBB: the trojan horse strategy of EVs

The BBB represents a formidable selective interface that preserves the neural environment from systemic influences. Constituted by brain microvascular endothelial cells (BMECs), this dynamic barrier, in concert with pericytes and astrocytes, composes the neurovascular unit (NVU), a complex sentinel that governs the cerebral ingress and egress of molecular entities. The discerning permeability of BBB is pivotal for neural homeostasis but simultaneously poses significant impediments to the pharmacotherapy of neurodegenerative disorders, thwarting the passage of most therapeutic agents [129, 130].

However, EVs with their innate ability to traverse biological membranes emerge as a ‘Trojan Horse’, deftly navigating the BBB. Recent empirical evidence underscores the heterogeneous penetrative capacities of EVs across this barrier. A recent investigative delineated the trans-BBB trafficking of various EV populations, discerning a spectrum of permeability across mouse, human, neoplastic, and non-neoplastic cellular lineages, thereby illuminating the differential crossing efficacies that could be harnessed therapeutically [131].

The precise modalities by which EVs infiltrate the NVU remain an area of intense research. It is posited that transcellular transport of EVs ensues via endocytic uptake by BMECs, an event capable of instigating phenotypic alterations within these cells and modulating the selective permeability of BBB [25, 132, 133]. This is exemplified by studies demonstrating that inducible pluripotent stem cell-derived small EVs (iPSC-sEVs) appear to counteract senescence in the BBB, offering cytoprotection in ischemic cerebrovascular insults [134]. Further elucidating the potential of EVs, it has been shown that MSC-derived EVs, as well as those from brain endothelial origins, can fortify the integrity of the BBB by modulating the Caveolin-1-dependent trafficking of tight junction proteins, such as ZO-1 and Claudin-5, thus preserving barrier function during acute ischemic events [135].

In addition to transcellular routes, EVs may exploit paracellular pathways, transiting via the inter-endothelial junctional complexes under certain physiological conditions [128]. Conversely, pathological derangements in the BBB, such as those encountered in NDD states, may precipitate a heightened permeability that permits the ingress of neurotoxic EVs, exacerbating neuronal injury [129]. This dualistic impact of EVs on the BBB, either as vehicles of repair or as conveyors of damage, heralds a nuanced understanding of their role in CNS pathobiology.

EVs represent a versatile arsenal in the quest to overcome the pharmacological sanctuary imposed by the BBB. The ability of EVs to interface with the NVU and modulate its properties not only underscores their potential as delivery vectors for therapeutic agents but also as modifiers of barrier physiology with implications for disease modulation. Further elucidation of the mechanisms guiding EV translocation and the subsequent effects on the NVU will enhance our capacity to exploit these nanovesicles for therapeutic gain.

### Biogenesis of therapeutically competent EVs: potential cellular factories

The therapeutic deployment of EVs in NDD management necessitates an intricate understanding of their biogenesis and the cellular provenance that confers them with a neuroprotective and regenerative prowess. MSCs, by their inherent propensity to secrete EVs laden with reparative molecules, stand at the forefront of potential cellular factories. Their autologous use in clinical settings harnesses this natural reservoir of therapeutic agents, offering a promising avenue for the amelioration of NDDs [63, 136].

The landscape of potential cellular sources for therapeutic EVs extends beyond MSCs. Dendritic cells (DCs), Mϕ, and neutrophils, including neutrophil-like promyelocytic cells, are increasingly recognized for their contributory roles in this context. DC-derived EVs, pivotal in the orchestration of immune responses, also partake in antigen presentation, a function that might be exploitable for disease-modifying strategies in NDDs [137–139]. Of particular interest are EVs from immature DCs, which are proficient in the release of EVs devoid of T-cell co-stimulatory molecules, such as major histocompatibility complex class II (MHC-II), thereby proffering an immunologically quiescent profile for therapeutic purposes [140].

Monocytes and Mϕ represent another cadre of cellular factories, capable of generating EVs that can elude the reticuloendothelial system, thereby circumventing one of the major barriers to effective drug delivery [138]. Such an attribute not only enhances the direct transport of therapeutic cargoes to targeted cells but also augments the therapeutic index of encapsulated drugs [139].

Moreover, neutrophils and their progenitors, the promyelocytes, are emerging as prolific producers of EVs. Their ability to rapidly secrete large quantities of EVs, coupled with their innate biological functions, positions them as a valuable source for the generation of EVs tailored for drug delivery. The drug-delivery systems derived from such EVs could potentially leverage the natural navigation systems of neutrophils, which instinctively migrate towards inflammation sites, a common feature of NDD pathogenesis.

Collectively, these cellular sources offer a palette of biogenetic origins for the derivation of therapeutically competent EVs. The selection of specific cellular factories for EV production must consider not only the innate characteristics of the EVs they produce but also the pathophysiological context of the NDD in question. Understanding the nuances of EV biogenesis from these diverse cellular origins will advance our capacity to tailor EV-based therapies to the complex demands of NDD intervention. Such advancements will pave the way for more precise and effective treatments, harnessing the full potential of EVs as carriers of regenerative medicine.

### Bioengineering EVs for precision targeting of CNS pathology

The precise targeting of CNS pathologies using EVs represents a paradigm shift in the therapeutic approach to NDDs. However, the inherent limitations of natural EVs, namely their suboptimal targeting efficiency, limited circulation half-life, and variable therapeutic payload delivery, necessitate the application of advanced bioengineering techniques to enhance their functionality and therapeutic precision.

The modification of EV surface ligands represents a critical frontier in this effort [141]. Utilizing innovative bioengineering techniques, researchers have sought to augment the innate homing capabilities of EVs. A pioneering approach by Alvarez-Erviti et al. demonstrated the potential of such modifications, where DCs were engineered to express a chimeric protein consisting of the lysosome-associated membrane protein 2b (Lamp2b) fused with a neuron-specific rabies viral glycoprotein (RVG) peptide. The EVs resulting from this molecular alteration have a heightened affinity for neuronal tissues, subsequently increasing their accumulation in regions pivotal for neurodegenerative processes, such as the cerebral cortex and hippocampus [137]. Further refinements in this domain have been illustrated by Cui et al., who showed that MSC-derived EVs (MSC-EVs) modified with the RVG peptide not only preferentially homed to neural tissue but also conferred notable anti-inflammatory effects in an AD mouse model. These bioengineered MSC-RVG-EVs downregulated pro-inflammatory cytokines, upregulated anti-inflammatory IL-10, and importantly, improved cognitive function, which is a significant therapeutic outcome [142].

Beyond surface engineering, conditioning of parental cells under specific culture conditions has emerged as a potent strategy to influence the biological activity of secreted EVs. For instance, AD-MSCs cultured in neurogenesis-promoting media have been shown to yield EVs with enhanced neural differentiation potential, indicating that the microenvironmental conditioning of progenitor cells can significantly impact the therapeutic profile of EVs [125]. Moreover, the microenvironmental oxygen tension has been implicated in the modulation of EV function. Studies by Huang et al. revealed that AD-MSCs subjected to hypoxic conditions not only exhibited increased EV secretion but also an enriched expression of specific microRNAs like miR-511-3p, known for their anti-inflammatory properties [143]. Additionally, there has been growing interest in utilizing advanced bioreactor technologies to scale up EV production. Jeske et al. reported that culturing human MSCs (hMSCs) in a 3D dynamic bioreactor significantly boosted the yield of EVs and enhanced the profile of their therapeutic cargo, suggesting that bioprocessing strategies can be pivotal in optimizing EV-based therapies for clinical applications [144].

The cumulative advancements in the bioengineering of EVs hold the promise of creating precision-targeted therapeutic agents capable of navigating the complex biological environment of the CNS. By leveraging these sophisticated methodologies, there is a realizable prospect of developing a new class of EV-based interventions that can address the intricate challenges posed by neurodegenerative pathologies with unprecedented specificity and efficacy.

### The Future of drug delivery: EVs in the clinical setting

The landscape of therapeutic strategies for NDDs is on the cusp of a significant paradigm shift with the advent of EVs as drug delivery systems (DDS) [145]. Nanotechnology has paved the way for innovative approaches aimed at traversing the complex environment of the CNS. However, the functional attributes of any DDS, including biodistribution and drug release profiles, are inherently dependent upon the physicochemical properties of the carrier system [128].

Traditional DDS such as polymeric nanoparticles and liposomes have been explored extensively, with both modalities revealing unique advantages and inherent limitations. Polymers like polycaprolactone (PCL), polylactic acid (PLA), and poly(lactic-co-glycolic acid) (PLGA), despite their versatility, are often challenged by suboptimal drug loading efficiencies and potential localized immune responses [129, 146]. Liposomes, with their biocompatible phospholipid constructs, offer advantages in drug encapsulation but are not exempt from issues like off-target effects and rapid clearance from circulation [129].

EVs, by contrast, emerge as endogenously derived vectors that are inherently biocompatible, low in immunogenicity, and proficient at crossing the BBB. This facilitates a targeted delivery mechanism that is both specific to the neural tissue and favorable in terms of reduced systemic toxicity [128, 147, 148]. The global research community is actively unraveling the potential of EVs in this domain.

The structural integrity of EVs allows for prolonged storage at cryogenic temperatures with minimal degradation, ensuring the stability of encapsulated therapeutic agents ranging from enzymes to nucleic acids [148, 149]. This has led to successful encapsulation and delivery of therapeutically active molecules like the CB2 receptor agonist AM1241, which exhibits enhanced brain bioavailability and therapeutic efficacy when delivered via MSC-EVs [150]. Similarly, bioactive compounds such as quercetin and curcumin, as well as protein modulators like tyrosine phosphatase-2 (SHP2), have been efficiently packaged within EVs, providing a therapeutic thrust against hyperphosphorylated tau and mitochondrial dysfunction associated with AD [151–153]. For PD, catalase and dopamine have been loaded in EVs to attenuate neuroinflammation and replenish neurotransmitter levels respectively [138, 154].

RNA-based therapeutics have garnered interest for their capacity to modify disease progression. Engineered EVs carrying BACE1-targeted siRNA have demonstrated a potent capacity to mitigate Aβ synthesis by significantly suppressing BACE1 expression in vivo [137, 155]. Further, miRNA-enriched EVs have been shown to exert neuroprotective effects, with miR-29 and miR-22 variants downregulating pathological Aβ production and inflammatory cascades in AD models [156, 157]. Additionally, m6A modifications in RNA processes, implicated in PD, have been targeted using MSC-EVs to deliver demethylase FTO-targeted siRNAs, demonstrating neuroprotective outcomes [158].

We have encapsulated this burgeoning field in Table 2, summarizing the prospective therapeutic EVs, their cellular origins, and their bioactive constituents. Mirroring the swift advancements in mRNA-based therapeutics for infectious diseases and oncology, the prospective integration of mRNA drugs into EV platforms for NDD treatment holds immense promise [159]. This burgeoning frontier of EV-based mRNA delivery could potentially redefine the therapeutic approach to NDDs, offering a vehicle of unprecedented specificity and reduced immunogenicity in the clinical armamentarium against these intractable conditions.

**Table 2:** Summary of EVs administration for NDDs

Original cells or tissues	Cargo	Function	Disease	Reference
Mϕ	Curcumin	Inhibit hyperphosphorylation of tau	AD	[152]
Blood plasma	Quercetin	Inhibit phosphorylation of tau	AD	[151]
AD-MSCs, BM-MSCs	NEP	Degrade Aβ	AD	[94, 160]
BM-MSCs	SHP2	Alleviate mitochondrial damage-mediated apoptosis	AD	[153]
BM-MSCs	CB2 receptor agonist (AM1241)	Facilitate Aβ phagocytosis and promote neurogenesis	AD	[150]
DCs	BACE1 siRNAs	Knockdown of BACE1	AD	[137]
BM-MSCs, HEK-293T cells	miR-29	Downregulate BACE1	AD	[156]
AD-MSCs	miR-22	Inhibit apoptosis and ameliorate neuroinflammation	AD	[157]
Fresh serum	Dopamine	Increase dopamine distribution in brain	PD	[154]
Mϕ, BM-MSCs	Catalase	Antioxidant effect	PD, AD	[138, 161]
Mϕ	GDNF	Ameliorate neuroinflammation	PD	[162]
Human umbilical cord-derived MSCs	m6A demethylase FTO-targeted siRNAs	Alleviate dopaminergic neuronal death	PD	[158]
Trophoblast- derived MSCs	miR-100a-5p	Antioxidant effect	PD	[163]
hBM-EPCs	Angiogenic factors	Ameliorate impaired brain endothelial cells	ALS	[67]
AD-MSCs	let-7b, miR-9, miR-124	Promote neurogenesis	-	[125]

*Abbreviations*: AD: Alzheimer’s disease, Aβ: amyloid-β peptides, PD: Parkinson’s disease, ALS: Amyotrophic lateral sclerosis, DCs: dendritic cells, Mϕ: macrophages, MSCs: mesenchymal stem cells, BM-MSCs: bone marrow-derived MSCs, AD-MSCs: adipose tissue-derived MSCs, NEP: Neprilysin, SHP2: tyrosine phosphatase-2, GDNF: glial cell-line derived neurotropic factor, hBM-EPCs: human bone marrow endothelial progenitor cells

## Conclusion and future prospects

**Fig. 5:**
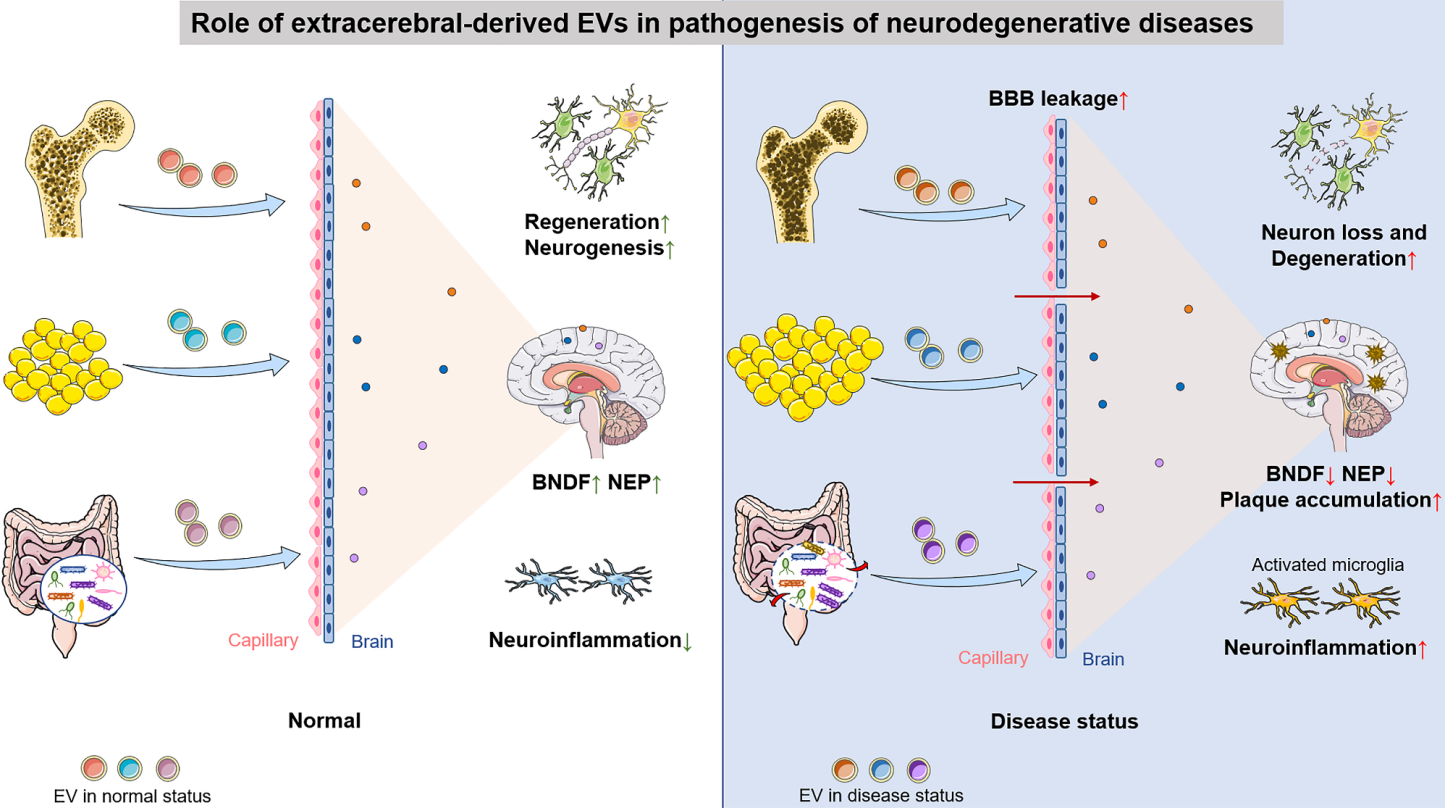
Role of extracerebral-derived EVs in pathogenesis of NDDs. This schematic delineates the dualistic influence of extracerebral-derived EVs on the progression of NDDs. In physiologically normal conditions, EVs originating from peripheral tissues such as bone, adipose, and the gut microbiome are instrumental in safeguarding cerebral function. Conversely, during pathological states like OP, metabolic syndrome, and gut dysbiosis, these extracerebral EVs facilitate a perturbation of the peripheral-brain axis. Such disturbances are implicated in neuronal attrition, degenerative changes, accumulation of neurotoxic species, and heightened neuroinflammatory responses, cumulatively exacerbating the pathogenesis of NDDs

In conclusion, this review provides a comprehensive overview of the dual role of extracerebral-derived EVs in the CNS and NDDs. We present new insights that, on the one hand, extracerebral-derived EVs have a protective effect on the brain in healthy states. On the other hand, with the occurrence of aging or disease, EVs derived from the periphery carry deleterious molecules that mediate disruption of the periphery-brain axis, which leads to the development of brain disorders such as NDDs (Fig. 5). This review has endeavored to distill the dichotomous nature of extracerebral-derived EVs within the neural landscape, elucidating their paradoxical roles as both custodians of cerebral integrity and potential harbingers of neurodegeneration. We have detailed the emerging paradigm where, in a state of homeostasis, extracerebral EVs contribute to the maintenance of brain health. In contrast, the advent of aging or pathologic states precipitates a shift, wherein these same vesicles become conveyors of detrimental cargo, effectuating dysregulation of the periphery-brain axis and potentially inciting the onset of NDDs (Fig. 5). Simultaneously, this narrative has spotlighted peripheral cell-secreted EVs as a novel and promising therapeutic modality for NDDs. On the one hand, the generation or release of harmful extracerebral EVs can be effectively suppressed through the use of specific protein inhibitors. One such inhibitor is the hyaluronic acid synthetase inhibitor, which has been shown to block the uploading of these detrimental EVs [23]. By treating the underlying peripheral diseases that drive the secretion of these harmful EVs, their secretion can also be significantly reduced. On the other hand, the secretion of beneficial EVs can be specifically stimulated or augmented. For instance, mechanical loading has been shown to revitalize osteocytes and enhance the secretion of EVs derived from them [164, 165]. This suggests that beneficial EVs may have the potential to be further optimized and their therapeutic efficacy enhanced through advanced engineering techniques. By harnessing these strategies simultaneously, it may be possible to achieve more significant therapeutic outcomes

Despite their potential, the trajectory toward the clinical application of EVs is fraught with complexities. The current landscape is limited by the absence of uniform protocols for EV isolation and a comprehensive understanding of their biogenetic and secretory machinery. To advance from bench to bedside, a rigorous framework for the optimization of EV-producing cell types is necessitated. This involves refining methodologies for cultivation, purification, and precise characterization of EVs. Moreover, it is imperative to undertake extensive safety evaluations, specifically in the context of long-term implications. The path to therapeutic implementation is one of incremental advancements. With each step, we gain deeper insights into the molecular mechanism of EV function and their interaction with neurobiological systems. In the future, it is envisioned that the integration of nanotechnology, bioengineering, and molecular biology will yield innovative platforms for the targeted delivery of EV-based therapies. In parallel, advances in omics technologies and systems biology promise to unravel the molecular signatures of EVs, fostering the development of bespoke therapeutic strategies

The prospective utility of EVs as vectors for precision medicine in NDDs holds immense promise. It beckons a new era of intervention strategies that leverage the innate biological synergy between EVs and the CNS. To actualize this potential, interdisciplinary collaboration and sustained investment in research are essential. The evolution of regulatory frameworks to accommodate these novel entities will be equally critical. As we chart this unexplored territory, the future beckons with the promise of harnessing the full therapeutic arsenal of EVs to confront the insidious challenge of NDDs.

## Data Availability

No datasets were generated or analysed during the current study.
